# High-level soccer players have a low rate of return to performance after hip arthroscopy for femoroacetabular impingement syndrome

**DOI:** 10.1007/s00167-023-07336-9

**Published:** 2023-03-22

**Authors:** Sofie Sjövall Anari, Alexander Olsson, Axel Öhlin, Neel Desai, Eric Hamrin Senorski, Mikael Sansone, Ida Lindman

**Affiliations:** 1 Department of Orthopaedics, Institute of Clinical Sciences, Sahlgrenska Academy, University of Gothenburg, Gothenburg, Sweden; 2 Department of Health and Rehabilitation, Institute of Neuroscience and Physiology, Sahlgrenska Academy, University of Gothenburg, Gothenburg, Sweden

**Keywords:** Femoroacetabular impingement syndrome, FAIS, Soccer, Return to sport, Return to performance, RTS, RTP, Elite, Sub-elite

## Abstract

**Purpose:**

Femoroacetabular impingement syndrome (FAIS) is a known cause of impaired sports performance in athletes and the relationship between FAIS and soccer players has previously been described. Hip arthroscopy is a viable treatment option that can facilitate athletes’ return to sport (RTS). The aim of this study was to evaluate the RTS and return to performance (RTP) with objective measurements in high-level soccer players after hip arthroscopy for FAIS.

**Method:**

Soccer players, with a hip sports activity scale (HSAS) level of 7 or 8 before symptom onset and undergoing hip arthroscopy for FAIS between 2011 and 2019 were identified in the Gothenburg hip arthroscopic registry. A total of 83 high-level soccer players, with a mean age of 23.9 (SD 4.4) years at surgery, were included. To verify the activity level and further stratify players as elite or sub-elite, player statistics were collected from soccer-specific scout webpages and the Swedish national soccer association. The return to sport was defined as return to one game of soccer. Return to performance was defined as playing at the same level, or higher, and participating in at least 80% of the number of games played the season before symptom onset or the season before surgery either the first or second season after hip arthroscopy.

**Results:**

In total, 71 (85.5%, 95% confidence interval (CI) 76.1–92.3%) of the players returned to sport the first or second season after surgery. Compared to the season before symptom onset, 31 (37.3%, 95% CI 27.0–48.7%) players returned to performance the first or second season after surgery, and 32 (38.6%, 95% CI 28.1–49.9%) players returned to performance the first or second season after surgery compared to the season before surgery.

**Conclusion:**

A high rate of elite and sub-elite soccer players return to soccer after hip arthroscopy for FAIS. However, less than half of the players RTP when evaluating performance through level of play and number of games played.

**Level of evidence:**

Level IV.

## Introduction

Femoroacetabular impingement syndrome (FAIS) is a well-known cause of hip pain and decreased range of motion in the hip joint, preventing athletes from pursuing their careers [10]. Femoroacetabular impingement syndrome is caused by abnormal anatomical morphologies, on the femur (cam morphology) and/or the acetabulum (pincer morphology), feasibly contributing to chondral and/or labral damages in the hip joint [4, 25]. When physiotherapy is not sufficient, hip arthroscopy is performed to restore the normal anatomy. Arthroscopic treatment contributes to gain of function, reduction of pain, and increases the probability of return to sport (RTS) [8, 9].

Soccer is a sport that requires changes of direction, hip flexion, adduction, and high rotational loads on the hip joint, all of which are movements that may provoke symptoms related to FAIS and the relationship between soccer and FAIS has been described in previous studies [1, 7, 13, 18]. The prevalence of FAIS varies depending on the population evaluated [8, 9, 11]. Falotico et al. [8] presented a prevalence of abnormal anatomy, radiographically, consistent with femoroacetabular impingement (FAI) in 93% of asymptomatic professional soccer players, compared to 28% in non-symptomatic, non-athletic controls. Heerey et al. [11] compared symptomatic soccer players with positive flexion-adduction-internal rotation (FADIR) test to asymptomatic soccer players with negative FADIR test and proposed cam morphology present radiographically in 71% and 63%, respectively.

An important question for soccer players undergoing hip arthroscopy for FAIS is whether they can return to their pre-injury level of sport, which may be difficult to answer since the definition of RTS varies between studies and has often been used as a binary outcome, not related to the pre-injury sports performance [6, 12, 27]. In previous studies, the RTS rate for athletes undergoing surgery for FAIS has varied greatly, from 56–100% [2, 5, 12, 20]. For example, Annin et al. [2] performed a systematic review and evaluated athletes from 18 different studies, who had played a sport for a minimum of two years and reported an RTS rate from 73–100%. While Ishøi et al. [12] evaluated 189 athletes performing different sports, through a self-reported return to sport questionnaire and reported that 108 athletes (57%) return to their pre-injury sport. However, several previous studies have calculated the RTS rate for athletes in general, not specifically soccer players, and the return to performance (RTP) rates have not been evaluated to the same extent as RTS [2, 5, 12, 21]. Furthermore, previous studies have often evaluated RTS with subjective data, received from for example surveys, where players responded with their interpretation [5, 12, 27]. Where measurement of RTS and RTP from objective data, such as players’ statistics, may increase the validity of the results. With increased knowledge and an increasing frequency of arthroscopy performed for FAIS, it is of great value for soccer players to obtain accurate information and reasonable expectations of RTS and RTP [3, 5]. The purpose of this study was to examine the RTS and RTP rates, through objective data, in high-level soccer players after hip arthroscopy for FAIS.

## Materials and methods

### Ethical approval

The study protocol was approved by the Swedish Ethical Review Authority (ID: 2019-06050 and ID: 2021-05412-02). Informed written consent was obtained from each participant before surgery.

### Participants

This is a prospective study evaluating high-level soccer players from the Gothenburg hip arthroscopic registry. The Gothenburg hip arthroscopic registry contains information about performed hip arthroscopies in two hospitals, Orthocenter Gothenburg and Mölndal/Sahlgrenska University Hospital [23]. The demographic data retrieved from the registry included duration of symptoms, operated side, FAI morphology, age, height, and weight [23].

**Fig. 1:**
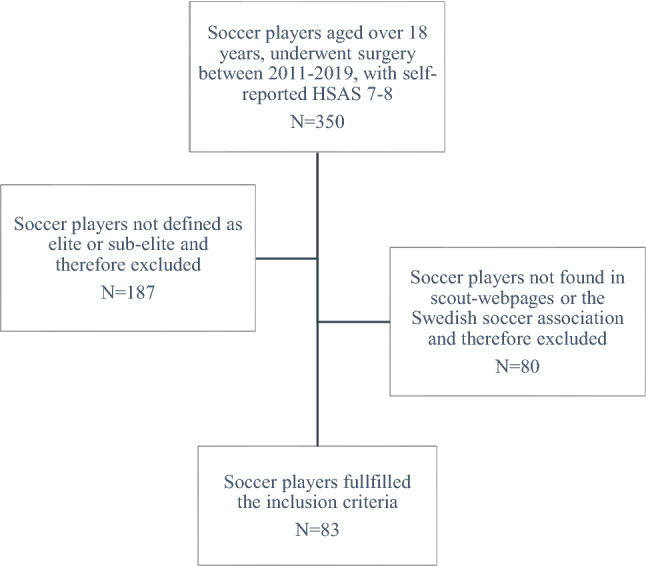
Flowchart. Flowchart of included soccer players

The inclusion criteria were self-reported sport of soccer, hip arthroscopic surgery for FAIS performed between 2011–2019, and age over 18 years at the time of surgery. A total of 83 soccer players (7 female and 76 male players) were included (Fig. 1). The mean age at onset of symptoms was 23.3 (SD 4.7) years and the mean age at surgery was 23.9 (SD 4.4) years. The median duration of symptoms before surgery was 12 months (range 1–108 months). All player characteristics are presented in Table 1. The included soccer players were further stratified as elite and sub-elite. The definition of elite soccer players in the present study corresponds to participation in the highest league in Sweden or a top-ranking international league, equivalent to the highest league in Sweden, the season before symptom onset, or the season before surgery. The definition of sub-elite soccer players corresponds to play in the second-highest league in Sweden the season before symptom onset or the season before surgery. The assortment was implemented by first assembling soccer players with a self-reported hip sports activity scale (HSAS) level of 7 or 8 before the onset of symptoms. The HSAS level 7–8 refers to athletes performing competitive sports, whereas for soccer players, HSAS 8 represents an elite level and HSAS 7 refers to athletes in minor or collegiate leagues [19]. To further stratify soccer players as elite or sub-elite and to verify the players performance status, publicly available statistics from the soccer-specific scout webpages (https://football.instatscout.com/ and https://app.scoutpanel.com/), as well as the Swedish national soccer association, were used. The data from soccer-specific scout webpages and the Swedish national soccer association, regarding the soccer player’s career, included on-field position, level of play, and the number of games played each evaluated season. The player positions were grouped as forward, midfield, defense, and goalkeeper. The level of play and number of games played from the season before the symptom onset and the season before surgery was compared to the first and second seasons played after hip arthroscopic surgery. Exclusion criteria were retirement from soccer before hip arthroscopy or if they did not meet the criteria for the definition of elite or sub-elite the season before the symptom onset or the season before surgery. If a player or data from the seasons before symptom onset or before surgery were not found on the soccer-specific scout webpages or the Swedish national soccer association database, this was considered as the player had not played at an elite or sub-elite level, and the player was therefore excluded. A total of 350 soccer players were found in the Gothenburg hip arthroscopic registry, 187 players were excluded for not reaching the definition of elite or sub-elite and 80 were not found in scout webpages or the Swedish soccer association and therefore excluded (Fig. 1).

**Table 1:** Player characteristics

Variable value (*n* = 83)	
Age at symptom debut, years	23.3 (SD 4.7)/23 (range 15–36)
Age at surgery, years	23.9 (SD 4.4)/23 (range 18–38)
Sex	
Male	76 (91.6%)
Female	7 (8.4%)
BMI, kg/m^2^	23.3 (SD 1.6)/24.5 (range 19.4–26.6)
Symptom duration, months	20.1 (SD 18.0)/12 (range 1–108)
Morphology	
Cam	26 (31.3%)
Pincer	0
Mixed	57 (68.7%)
Bilateral surgery	54 (65.1%)
Position	
Forward	27 (32.5%)
Midfield	23 (27.7%)
Defender	9 (10.8%)
Goalkeeper	1 (1.2%)
Missing data	23 (27.7%)

Data are reported as mean (standard deviation)/median (range), or *n* (%)

*BMI* body mass index

### Indications for surgery

All included soccer players received the FAIS diagnosis through a triad of symptoms, clinical examination, and radiographic findings, as suggested by the Warwick agreement [10]. All soccer players had non-satisfying results with a previous non-surgical treatment such as physiotherapy and non-steroid anti-inflammatory drugs (NSAID). The arthroscopic surgical method has previously been described [22].

### Return to sport and performance

Return to sport was defined as participation in at least one game of soccer either the first or second season after hip arthroscopy. Return to performance was defined as playing at the same level, or higher, and participating in at least 80% of the number of games played the season before symptom onset or the season before surgery either the first or second season after hip arthroscopy. The calculation was performed for each player, therefore, each player acted as its own control. Both the level of play and the number of games were evaluated from the season before symptom onset and before surgery compared to the first and second season after hip arthroscopy. Since there, in team sports, might be a natural variation in the number of games played during one season, regardless of injury or not, a lower level than 100% was set for the definition of RTP. The variation was calculated from the variation in games played in a random set of players, without known medical history, from the highest league in Sweden the seasons of 2020 and 2021, a method previously described when evaluating RTS and RTP in ice hockey players [15]. In this current study it was found there could be a variation of 20% in games played between seasons, therefore, the limit for RTP was set at 80%.

### Statistical analysis

The distribution of continuous data are given with mean, SD, median, minimum, and maximum. Categorical data are given as numbers and percentages with exact 95% confidence intervals. To evaluate if there was a difference between the age at symptom onset, age when surgery was performed and RTS, the independent *t*-test was used since data were normally distributed. To evaluate if there was a difference between symptom duration and RTS, the Fisher non-parametric permutation test was used since data were not normally distributed. To evaluate the difference between RTS, RTP, and elite or sub-elite soccer players the Fisher exact test was used. All statistical tests were two-sided and conducted at the 5% significance level. All calculations were performed with IBM SPSS version 28 and SAS software 9.4.

## Results

**Fig. 2:**
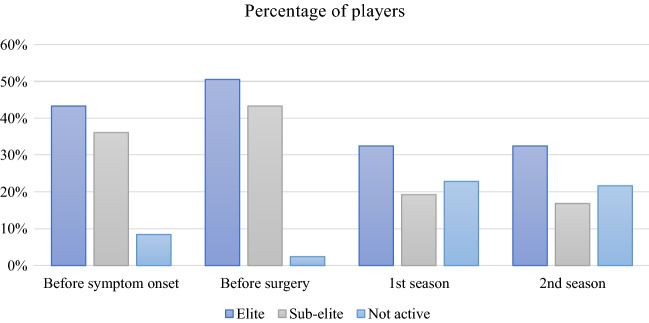
Percentage of players. The percentage of elite, sub-elite, and not active soccer players each season

During the season before symptom onset, the mean number of games played was 19.7 [(SD 10.6)/median 22 (range 1–47)], the season before surgery the mean number of games played was 21.7 [(SD 9.9)/median 23 (range 1–44)]. In the first and second season after surgery, the mean number of games played was 18.9 [(SD 10.7)/median 20 (range 1–41)] and 20.2 [(SD 10.9)/median 19 (range 1–45)], respectively. Of all included soccer players, 46 (55.4%) players were defined as elite and 37 (44.6%) as sub-elite. The season before symptom onset, 36 (43.4%) of the soccer players were defined as elite and 30 (36.1%) of the soccer players were defined as sub-elite. The season before surgery, 42 (50.6%) of the soccer players were defined as elite and 36 (43.4%) of the soccer players were defined as sub-elite (Fig. 2).

### Return to sport

In total, RTS, per definition participation in at least one game of soccer the first or second season after hip arthroscopy, was achieved by 71 (85.5%, 95% CI 76.1–92.3%) of the soccer players. In the first or second season after hip arthroscopy, RTS was achieved by 41 (89.1%, 95% CI 76.4–96.4%) of the soccer players defined as elite and by 30 (81.1%, 95% CI 64.8–92.0%) of the soccer players defined as sub-elite. In terms of RTS, there was no difference between elite or sub-elite players (n.s) (Table 2).

**Table 2:** Return to sports and return to performance

Outcome	All (n=83)	Elite (n=46)	Sub-elite (n=37)	Mean difference elite − sub-elite (95% CI) *p*-value
RTS 1- or 2-season after surgery *n* (%) (95% CI)	71 (85.5%) (76.1–92.3%)	41 (89.1%) (76.4–96.4%)	30 (81.1%) (64.8–92.0%)	8.0 (−9.9 –26.0) *p* = 0.47
RTP 1- or 2-season after surgery compared with the season before symptom onset *n* (%) (95% CI)	31 (37.3%) (27.0–48.7%)	20 (43.5%) (28.9–58.9 %)	11 (29.7%) (15.9–47.0%)	13.7 (−7.7 –33.9) *p* = 0.29
RTP 1- or 2-season after surgery compared with the season before surgery *n* (%) (95% CI)	32 (38.6%) (28.1–49.9%)	20 (43.5%) (28.9–58.9%)	12 (32.4 %) (18.0 – 49.8 %)	11.0 (− 12.2 –34.3) *p* = 0.42

Overview of return to sports and return to performance for total and by elite/sub-elite

*CI* confidence interval

Statistical significance was set at *p* < 0.05

There were no differences with regard to age at symptom debut (n.s) nor age when surgery was performed (n.s) and RTS (Table 3). However, a significant difference was found regarding symptom duration and RTS (*p* = 0.018). Players that did not RTS had a longer symptom duration before surgery [mean 32.6 months (SD 28.4)/median 24 months (range 6–108 months)] compared to soccer players who did RTS [mean 17.8 months (SD 14.7)/median 12 months, (range 1–60 months)] (Table 3).

**Table 3:** Baseline characteristics by return to sports and by return to performance

Groups	Return to sport			Return to performance		
	Yes	No	Mean difference (95% CI) *p*-value	Yes	No	Mean difference (95% CI) *p*-value
Age at symptom debut (years)	23.3 (4.5 SD) 23.0 (15.0;34.0)	23.8 (6.1 SD) 22.0 (15.0;36.0)	0.5 (− 2.5–3.4) *p* = 0.75	23.0 (3.7 SD) 22.0 (16.0;32.0)	23.6 (5.4 SD) 23.0 (15.0;36.0)	0.6 (− 1.5–2.7) *p* = 0.55
Age at surgery (years)	23.8 (4.2 SD) 23.0(18.0;34.0)	24.7 (5.9 SD) 22.5 (18.0;38.0)	0.9 (− 1.9–3.6) *p* = 0.53	23.5 (3.5 SD) 23.0 (18.0;32.0)	24.3 (5.0 SD) 23.0 (18.0;38.0)	0.8 (− 1.2–2.7) *p* = 0.44
Symptom duration (months)	17.8 (14.7 SD) 12.0 (1.0;60.0)	32.6 (28.4 SD) 24.0 (6.0;108.0)	14.8 (2.8–24.7) *p* = 0.018	15.9 (14.0 SD) 12.0 (2.0;60.0)	23.0 (20.1 SD) 18.0 (1.0;108.0)	7.16 (− 0.9–15.3) *p* = 0.082

Continuous variables are presented as mean (standard deviation)/median (min;max)

*CI* confidence interval

Statistical significance was set at *p* < 0.05

### Return to performance

When evaluating RTP, per definition participation in at least 80% of the number of games at the same, or higher level, it was achieved by 31 (37.3%, 95% CI 27.0–48.7%) soccer players the first or second season after surgery compared to the season before symptom onset. Of the players who did RTP, 20 (24.1%, 95% CI 15.4–34.7%) returned within the first season after surgery. A total of 20 (43.5%, 95% CI 28.9–58.9%) elite soccer players and 11 (29.7%, 95% CI 15.9–47.0%) sub-elite soccer players achieved RTP the first or second season after surgery compared to the season before symptom onset (Table 2). When comparing the season before surgery to the first or second season after surgery, 32 (38.6%, 95% CI 28.1–49.9%) of the soccer players RTP and 19 (22.9%, 95% CI 14.4–33.4%) of these players returned within the first season after surgery. A total of 20 (43.5%, 95% CI 28.9–58.9%) elite and 12 (32.4 %, 95% CI 18.0–49.8%) sub-elite soccer players achieved RTP the first or second season after surgery compared to the season before surgery. In terms of RTP, there was no significant difference between elite or sub-elite soccer players (n.s) (Table 2).

When comparing the season before symptom onset to the first season after surgery, 43 (51.8%, 95% CI 40.6–62.9%) of the soccer players played at the same or higher level, and just as many played at the same or higher level when comparing the season before surgery to the first season after surgery.

## Discussion

The most important finding of the present study was that a high rate, 85.5%, of soccer players defined as elite or sub-elite, RTS after hip arthroscopy for FAIS. However, less than half of the players RTP. Of the soccer players who RTP, the majority returned during the first season after surgery.

Only a handful of previous studies have examined soccer players rate of RTS and RTP after arthroscopic surgery for FAIS. Locks et al. [17] used webpages to evaluate soccer players number of games pre-and postoperative and reported that 96% of soccer players returned to play, however, no further specification of the definition of return to play could be found. Casartelli et al. [5] performed a systematic review evaluating athletes in general, where on average 87% RTS, and 82% returned to the same level of sport. Unfortunately, only a small proportion of the athletes were soccer players, and the majority of the included studies had an ambiguous definition of RTS, as 18 articles were reviewed and there was no mutual definition of RTS [5]. The result in this study is slightly lower for RTS than in previous studies, which may be the result of the objective definition of RTS used, where only statistics from performed games were evaluated [5, 17, 27]. Several studies [12, 27] have evaluated RTS and RTP in athletes according to the consensus statement by Arden et al. [3]. Wörner et al. [27] evaluated RTS and RTP in different types of athletes, by asking if the athletes had returned to sport and performance through an online survey and reported that 89% RTS and 21% returned to their pre-symptom level of performance [27]. Ishøi et al. [12] did likewise evaluate RTS in athletes in different sports, at elite-, competitive- and recreational levels, through a web-based survey and suggested 57% return to their pre-injury sport at pre-injury level, yet only 17% reported optimal sports performance [12]. Although both previous studies [12, 27] and the current study have evaluated athletes RTS and RTP in accordance with the consensus statement by Arden et al. [3], different methods for measuring the rate of RTS and RTP were used and, therefore, the results might differ [12, 27]. However, a part of the difference may again be due to the difference in athletes evaluated and the definition of return. In several previous studies [5, 12, 17, 27], an evaluation of return to the level of play and sport has been made, however, in this current study, the RTP was calculated by objective measures, comparing the level of play in combination with games played during the season before symptom onset and before surgery to the first and second season after surgery. This could possibly explain the different results in terms of RTP. The result highlights the common challenge concerning the evaluation of RTS and RTP after hip arthroscopy for FAIS. Definitions are often imprecise or subjectively measured and differs between studies, which complicates the comparison of results [6]. When subjective measures are used and the athletes themselves register the return to sport or performance, the athletes can, theoretically, over- or underestimate their return. An athlete can return to training after surgery and still register a return to sport or performance. In those studies, the reported data on RTS may therefore be falsely high.

On the other hand, studies rarely report reasons for not reaching RTS or RTP, potentially leading to low values, for example, if a player had planned to retire before surgery. In the current study, a small proportion of soccer players returned to a lower level of play post-surgery yet played the same number of games or more compared to the season before symptom onset or before surgery. As per the definitions in this study, these players did RTS, however, not RTP. Professional athletes are probably more interested in information regarding RTP than RTS and the fact that there might be a difference between RTS and RTP can have a major impact on athletes in the preoperative discussions regarding surgery and patient expectations. A return to pre-injury sport does not in all cases mean a return to optimal sports performance and satisfaction [12]. Unfortunately, there was no consideration of the reasons why soccer players did not RTS or RTP in this study, which might lead to falsely low values of RTP. Even if the evaluation of both RTS and RTP through objective measures, such as player statistics, can contribute to more reasonable expectations prior to a hip arthroscopy for FAIS, there are several possible reasons for not RTS or RTP, such as persistent hip pain, age, socioeconomics, or the asset of external support [14, 26].

A significant difference was found between RTS and symptom duration, where soccer players who did not RTS had a longer symptom duration. This confirms results from previous studies, which have reported an association between longer symptom duration, poorer outcomes, and lower levels of RTS [16, 22, 24]. In the current study, no evaluation was made with regard to the soccer players intraarticular cartilage damage. However, according to previous studies, longer symptom duration can lead to more chondral damage and onset of osteoarthritis, making the joint more responsive to high loads and thereby affecting athletes ability to RTS, which potentially could explain the association found [22, 24].

This study is not without limitations. Data on soccer players positions were not analyzed despite data collection as there were missing data for 28% of the soccer players. Another limitation is that no power analysis was performed, however, all who met the inclusion criteria were included. This study evaluated 350 soccer players, of which 187 soccer players played at levels too low to be defined as elite or sub-elite. This could mean the players wrongly classified their activity level as HSAS 7–8, which makes one question the validity of self-reported activity level. Furthermore, 80 soccer players could not be found on scout webpages or the Swedish national soccer association, which could mean the registry has missing data from the period the players were active or the players might be active in a foreign league not available for us. This is, however, improbable in a large registry with high-level soccer players. To decrease the risk of self-reported bias, it was decided that if the database did not possess information from the season planned to be evaluated, the player was defined as not active in elite or sub-elite level.

Even if the field of FAIS has grown exceptionally in the past decade, much remains to be clarified. It is desirable to find a more adequate method to evaluate athletes level of play and to compose an unambiguous and shared definition of RTS and RTP. The results of this study are of importance when discussion to perform surgery begins, to provide soccer players and other stakeholders in soccer with more knowledge on the outcome and reasonable expectations following hip arthroscopy for FAIS. Especially since the rates of RTS and RTP have varied greatly.

## Conclusion

When evaluating RTS and RTP after hip arthroscopy for FAIS with objective measurements, elite and sub-elite soccer players have a high rate of RTS, where almost 9 out of 10 players return. However, less than half of the players RTP.

## Data Availability

The data that support the findings of this study are available from https://football.instatscout.com/, https://app.scoutpanel.com/ and the Swedish national soccer association. Regarding data from the Swedish national soccer association, restrictions apply to the availability of these data and so these are not publicly available. Data are however available from the authors upon reasonable request and with permission from the Swedish national soccer association.

## Abbreviations

FAI: Femoroacetabular impingement
FAIS: Femoroacetabular impingement syndrome
FADIR: Flexion-adduction-internal rotation
HSAS: Hip sports activity scale
RTP: Return to performance
RTS: Return to sport

## References

[1] Agricola R, Bessems JH, Ginai AZ, Heijboer MP, van der Heijden RA, Verhaar JA (2012). The development of Cam-type deformity in adolescent and young male soccer players. Am J Sports Med.

[2] Annin S, Lall AC, Yelton MJ, Shapira J, Rosinsky PJ, Meghpara MB (2021). Patient-reported outcomes in athletes following hip arthroscopy for femoroacetabular impingement with subanalysis on return to sport and performance level: a systematic review. Arthroscopy.

[3] Ardern CL, Glasgow P, Schneiders A, Witvrouw E, Clarsen B, Cools A (2016). 2016 Consensus statement on return to sport from the First World Congress in Sports Physical Therapy, Bern. Br J Sports Med.

[4] Beck M, Kalhor M, Leunig M, Ganz R (2005). Hip morphology influences the pattern of damage to the acetabular cartilage: femoroacetabular impingement as a cause of early osteoarthritis of the hip. J Bone Joint Surg Br.

[5] Casartelli NC, Leunig M, Maffiuletti NA, Bizzini M (2015). Return to sport after hip surgery for femoroacetabular impingement: a systematic review. Br J Sports Med.

[6] Chona DV, Bonano JC, Ayeni OR, Safran MR (2020). Definitions of return to sport after hip arthroscopy: are we speaking the same language and are we measuring the right outcome?. Orthop J Sports Med.

[7] Doran C, Pettit M, Singh Y, Sunil Kumar KH, Khanduja V (2022). Does the type of sport influence morphology of the hip? A systematic review. Am J Sports Med.

[8] Falotico GG, Arliani GG, Yamada AF, Fernandes A, Ejnisman B, Cohen M (2019). Professional soccer is associated with radiographic cam and pincer hip morphology. Knee Surg Sports Traumatol Arthrosc.

[9] Gerhardt MB, Romero AA, Silvers HJ, Harris DJ, Watanabe D, Mandelbaum BR (2012). The prevalence of radiographic hip abnormalities in elite soccer players. Am J Sports Med.

[10] Griffin DR, Dickenson EJ, O’Donnell J, Agricola R, Awan T, Beck M (2016). The Warwick Agreement on femoroacetabular impingement syndrome (FAI syndrome): an international consensus statement. Br J Sports Med.

[11] Heerey J, Agricola R, Smith A, Kemp J, Pizzari T, King M (2021). The size and prevalence of bony hip morphology do not differ between football players with and without hip and/or groin pain: findings from the FORCe cohort. J Orthop Sports Phys Ther.

[12] Ishøi L, Thorborg K, Kraemer O, Hölmich P (2018). Return to sport and performance after hip arthroscopy for femoroacetabular impingement in 18- to 30-year-old athletes: a cross-sectional cohort study of 189 athletes. Am J Sports Med.

[13] Knapik DM, Gaudiani MA, Camilleri BE, Nho SJ, Voos JE, Salata MJ (2019). Reported prevalence of radiographic cam deformity based on sport: a systematic review of the current literature. Orthop J Sports Med.

[14] Ko SJ, Terry MA, Tjong VK (2020). Return to sport after hip arthroscopy for femoroacetabular impingement syndrome: a comprehensive review of qualitative considerations. Curr Rev Musculoskelet Med.

[15] Lindman I, Löfskog M, Öhlin A, Abrahamsson J, Hamrin Senorski E, Karlsson J (2022). Return to sport for professional and subelite ice hockey players after arthroscopic surgery for femoroacetabular impingement syndrome. Orthop J Sports Med.

[16] Lindman I, Öhlin A, Desai N, Samuelsson K, Ayeni OR, Hamrin Senorski E (2020). Five-year outcomes after arthroscopic surgery for femoroacetabular impingement syndrome in elite athletes. Am J Sports Med.

[17] Locks R, Utsunomiya H, Briggs KK, McNamara S, Chahla J, Philippon MJ (2018). Return to play after hip arthroscopic surgery for femoroacetabular impingement in professional soccer players. Am J Sports Med.

[18] Marom N, Dooley MS, Burger JA, Chang B, Coleman SH, Ranawat AS (2020). Characteristics of soccer players undergoing primary hip arthroscopy for femoroacetabular impingement: a sex- and competitive level-specific analysis. Am J Sports Med.

[19] Naal FD, Miozzari HH, Kelly BT, Magennis EM, Leunig M, Noetzli HP (2013). The Hip Sports Activity Scale (HSAS) for patients with femoroacetabular impingement. Hip Int.

[20] Parvaresh KC, Wichman D, Rasio J, Nho SJ (2020). Return to sport after femoroacetabular impingement surgery and sport-specific considerations: a comprehensive review. Curr Rev Musculoskelet Med.

[21] Reiman MP, Peters S, Sylvain J, Hagymasi S, Mather RC, Goode AP (2018). Femoroacetabular impingement surgery allows 74% of athletes to return to the same competitive level of sports participation but their level of performance remains unreported: a systematic review with meta-analysis. Br J Sports Med.

[22] Sansone M, Ahldén M, Jonasson P, Thomeé C, Swärd L, Baranto A (2015). Good results after hip arthroscopy for femoroacetabular impingement in top-level athletes. Orthop J Sports Med.

[23] Sansone M, Ahldén M, Jonasson P, Thomeé C, Swärd L, Baranto A (2014). A Swedish hip arthroscopy registry: demographics and development. Knee Surg Sports Traumatol Arthrosc.

[24] Shibata KR, Matsuda S, Safran MR (2017). Arthroscopic hip surgery in the elite athlete: comparison of female and male competitive athletes. Am J Sports Med.

[25] Trigg SD, Schroeder JD, Hulsopple C (2020). Femoroacetabular impingement syndrome. Curr Sports Med Rep.

[26] Weber AE, Bolia IK, Mayfield CK, Ihn H, Kang HP, Bedi A (2021). Can we identify why athletes fail to return to sport after hip arthroscopy for femoroacetabular impingement syndrome? A systematic review and meta-analysis. Am J Sports Med.

[27] Wörner T, Thorborg K, Stålman A, Webster KE, Momatz Olsson H, Eek F (2018). High or low return to sport rates following hip arthroscopy is a matter of definition?. Br J Sports Med.

